# The Circular F-Actin Bundles Provide a Track for Turnaround and Bidirectional Movement of Mitochondria in *Arabidopsis* Root Hair

**DOI:** 10.1371/journal.pone.0091501

**Published:** 2014-03-13

**Authors:** Yu Zhang, Xiaojing Sheng, Xiangfei Meng, Yan Li

**Affiliations:** State Key Laboratory of Plant Physiology and Biochemistry, College of Biological Sciences, China Agricultural University, Beijing, China; University of Minnesota, United States of America

## Abstract

**Background:**

The movement of organelles in root hairs primarily occurs along the actin cytoskeleton. Circulation and “reverse fountain” cytoplasmic streaming constitute the typical forms by which most organelles (such as mitochondria and the Golgi apparatus) in plant root hair cells engage in bidirectional movement. However, there remains a lack of in-depth research regarding the relationship between the distribution of the actin cytoskeleton and turnaround organelle movement in plant root hair cells.

**Results:**

In this paper, *Arabidopsis seedlings* that had been stably transformed with a GFP-ABD2-GFP (green fluorescent protein-actin-binding domain 2-green fluorescent protein) construct were utilized to study the distribution of bundles of filamentous (F)-actin and the directed motion of mitochondria along these bundles in root hairs. Observations with a confocal laser scanning microscope revealed that there were widespread circular F-actin bundles in the epidermal cells and root hairs of *Arabidopsis* roots. In root hairs, these circular bundles primarily start at the sub-apical region, which is the location where the turnaround movement of organelles occurs. MitoTracker probes were used to label mitochondria, and the dynamic observation of root hair cells with a confocal laser scanning microscope indicated that turnaround mitochondrial movement occurred along circular F-actin bundles.

**Conclusions:**

Relevant experimental results demonstrated that the circular F-actin bundles provide a track for the turnaround and bidirectional movement of mitochondria.

## Introduction

The actin cytoskeleton is an important component for the establishment of polarity in plant cells [1]; in combination with myosin, the actin cytoskeleton promotes organelle movement within cells [2], [3]. One type of plant cell that typically exhibits high polarity and rapid apical growth is root hair cells. Studies have demonstrated that the polar growth of root hairs is closely related to the cytoskeleton of these cells [4], [5]. Thus, root hairs are an important model system for the study of organelle movement and the cytoskeleton in plant cells.

Investigations have determined that in root hair cells, actin filaments are primarily aligned in longitudinal or slightly helical bundles along the root hair axis [1], [6], [7]. To date, however, certain controversies remain unresolved regarding the arrangement of these filaments in the apical and sub-apical regions of root hairs. Observations of root hairs prepared through freezing and chemical fixation indicated the absence of F-actin bundles in a small region at the apex of these hairs [6], [8], and approaches that have used fluorescein isothiocyanate (FITC)-phalloidin microinjections or green fluorescent protein (GFP)-fused actin-binding proteins to label F-actin revealed only a modicum of disorganized filaments at the apex of *Arabidopsis* root hairs [9], [10]. However, other experiments have found that a cap-shaped accumulation of fibrous actin filaments occurs at the apex of root hairs [4], [11]–[13]. Observations of *Arabidopsis* root hairs labeled with GFP-fimbrin2 revealed dense bundles of F-actin parallel to the longitudinal axis of root hairs that were not present in the clear zone at the apex of these hairs [9], [10]. Employing an overall approach involving not only chemical fixation combined with vacuum infiltration but also the use of tetramethyl rhodamine isothiocyanate (TRITC)-phalloidin to label actin filaments in wheat root hairs, He et al. observed dense F-actin bundles along the long axis of root hairs; thin filaments from these dense sub-apical F-actin bundles branched and extended towards the apical regions of the root hairs [14].

Myosin family members are important molecular motor proteins in eukaryotic cells. Myosin proteins transport organelles, produce mechanical energy from ATP, and move along actin filaments. There are three main classes of plant myosin proteins: myosin VIII, myosin XI, and myosin XIII [15. 16]. In *Arabidopsis*, 17 myosin genes have been identified; four of these genes are members of the myosin VIII class, and the remaining 13 genes are members of the myosin XI class [17]. In evolutionary terms, the myosin XI class of plant genes is most closely related to the myosin V class of animal myosin genes [18]–[21], which are involved in the transport of organelles and vesicles. Studies have demonstrated that in plants, myosin XI proteins are involved in cytoplasmic streaming [22]. Subsequent in vitro examinations of myosin XI movements in higher plants exhibited the unique nature of the rapid movement of these myosins along actin filaments [23]. Using six types of yellow fluorescent protein (YFP)-myosin XI tail fusion proteins, Reisen and Hanson observed that the tails of myosin XI proteins can bind to specific vesicles and organelles [24]. By transiently expressing a protein in which GFP was fused to the head-neck domain of *Arabidopsis* MYA2 in the epidermal cells of a variety of plant species, Walter and Holweg found that the fusion proteins co-localized with cytoplasmic F-actin bundles [25], which was consistent with the rapid streaming of organelles. Through RNAi experiments, Avisar et al. demonstrated that in tobacco leaf cells, myosin XI-K was involved in the rapid trafficking of Golgi stacks, mitochondria, and peroxisomes [21]. Ojangu et al. found that *Arabidopsis* myosin XI-K influences root hair development and the trichome morphology of stems and leaves [26]. Peremyslov et al. used transferred DNA (T-DNA) insertion approaches to prove that *Arabidopsis* myosin XI-K and myosin XI-2 played important roles in root hair elongation and organelle transport [27].

During root hair growth, cytoplasmic flow and organelle movement primarily occur through circulation or “reverse fountain” streaming [28]. Studies have found that bundles of actin filaments are aligned in two directions in root hairs [29]. However, research is still required to determine how these bidirectionally oriented filament bundles are arranged and how turnaround organelle motion occurs in the sub-apical regions of root hairs.

## Results

### Circular F-actin Bundles Exist in *Arabidopsis* Root Hair Cells

**Figure 1:**
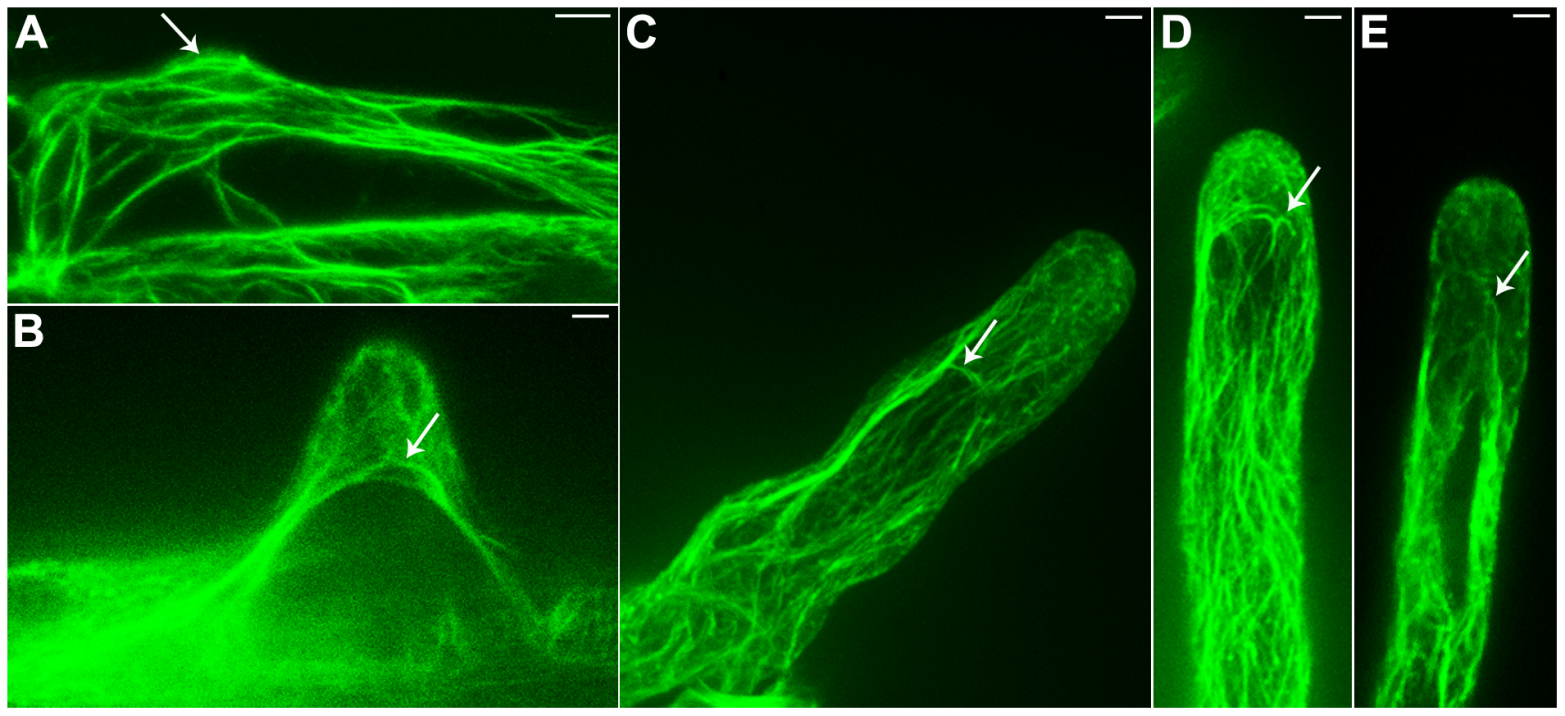
Confocal images of circular F-actin bundles in living root hairs of *Arabidopsis.* F-actin in living root hairs was labeled by GFP-ABD2-GFP which had a stable expression in *Arabidopsis*. (A) In short root hair tips, circular F-actin bundles were most close to the plasma membrane. (B–D) In the long root hairs, circular F-actin bundles were present in the sub-apical region. (E) At the sup-apex of the root hair, the angle of a few circular F-actin bundles turn around was smaller than that of A, B, C and D. *Arrows* indicate the circular F-actin bundles in sub-apical region of living root hairs. Bars = 5 µm.

**Figure 2:**
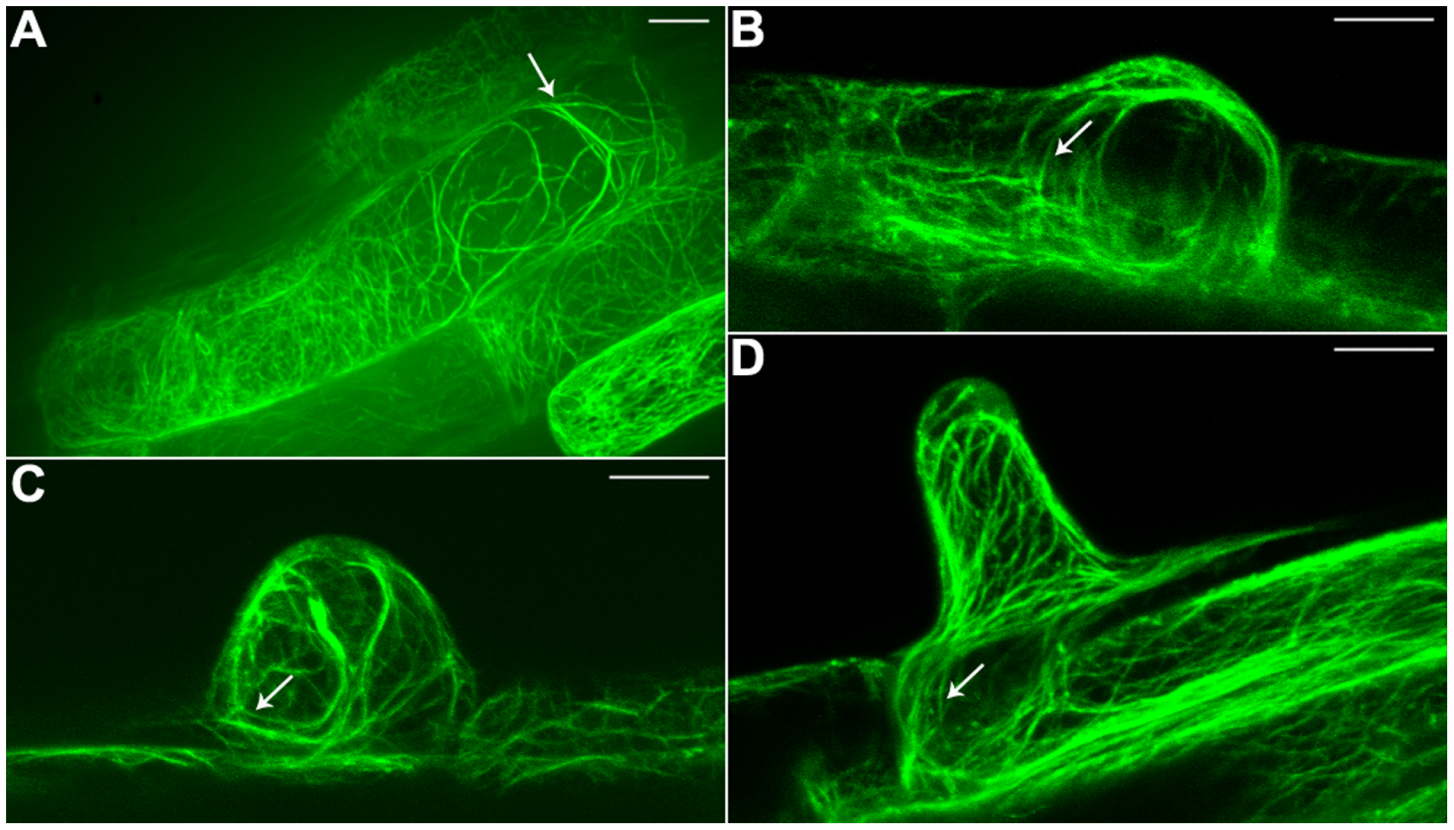
Confocal images of continuous circular F-actin bundles between root epidermal cells and root hairs. (A) Continuous circular F-actin bundles in root epidermal cell. (B–D) Continuous circular F-actin bundles between root epidermal cells and root hairs. *Arrows* indicate continuous circular F-actin bundles. Bars = 10 µm.

**Figure 3:**
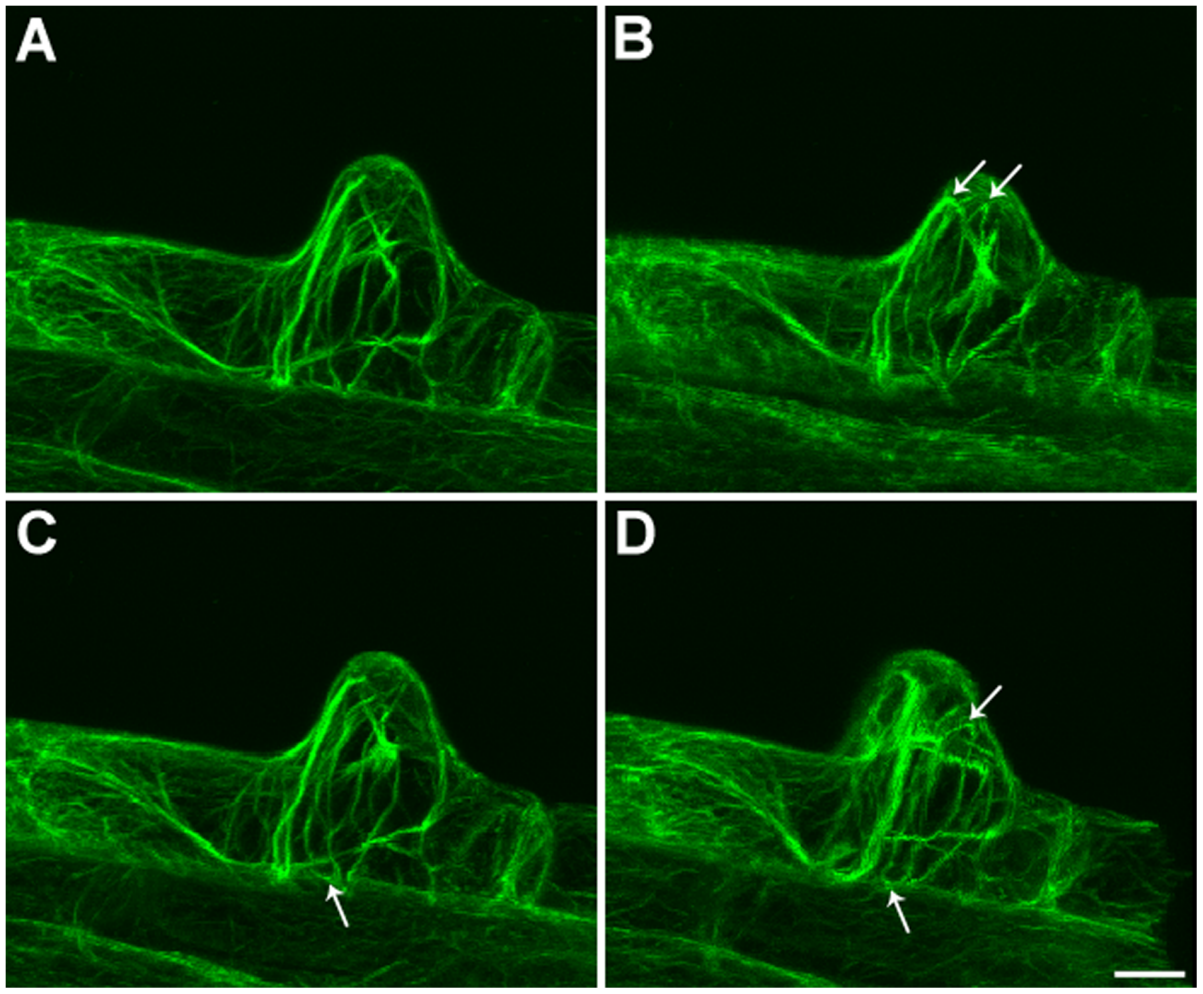
Confocal images of circular F-actin bundles between root epidermal cells and root hairs at different rotation angles in 3D reconstructions from higher resolution images. Original confocal image for GFP-ABD2-GFP stable expression root hair in *Arabidopsis*. (B) Circular F-actin bundles were observed at the sup-apex of the root hair after a rotation of 36° around the X axis (arrows). (C) Circular F-actin bundles were observed in the root epidermal cell after a 192° rotation around the X axis (arrow). (D) Circular F-actin bundles were observed between the root epidermal cell and root hair after a 324° rotation around the Y axis (arrows). Bar = 10 µm.

An *Arabidopsis* strain with stable expression of a GFP-actin-binding domain 2 (ABD2)-GFP fusion protein (provided by Elison B. Blancaflor) was utilized as the experimental material for this study. We found that the GFP-ABD2-GFP stable expression root hairs were similar to wild type root hairs in length and morphology (Figure S1). Observations of this material with a confocal microscope revealed that thick F-actin bundles were longitudinally aligned along the root hair, parallel to the direction of root hair elongation; this finding was consistent with the results of prior studies. In addition, we observed that thin filaments had branched out from the sub-apical F-actin bundles; these filaments extended to the apex of the root hairs, forming a network of thin filaments in the apical region of these root hairs (Figure 1C–D). This finding was consistent with the results that He et al. obtained using TRITC-phalloidin fluorescent labeling [14]. Moreover, we determined that prior to the apical clear zone, i.e., in the sub-apical regions of root hairs, circular F-actin bundles were formed by certain actin bundles that were distributed in a continuously turning manner (Figure 1, indicated by arrows). Elongating root hair cells of different lengths were observed, revealing that these circular F-actin bundles were present in the apical region of root hairs that have barely begun to grow (Figure 1A); during the apical growth of root hairs, the circular F-actin bundles in these root hairs extended towards the apical region. These bundles were always present prior to the apical clear zone, i.e., in the sub-apical region of root hairs (Figure 1B–E). In addition, we also found circular F-actin bundles in root epidermal cells (Figure 2A). The circular F-actin bundles formed a continuous ring of actin filaments between root epidermal cells and root hairs (Figure 2B–D). In 3D reconstructions from higher resolution images, at different rotation angles, we could find more circular F-actin bundles between root epidermal cells and root hairs (Fig. 3B–D).

### The Dynamic Observation of Circular F-actin Bundles in *Arabidopsis* Root Hairs

**Figure 4:**
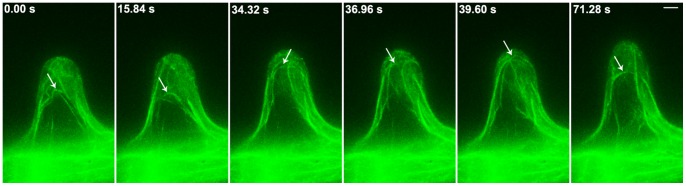
The Dynamic circular F-actin bundles in root hair of *Arabidopsis.* The confocal images were obtained for time-lapse scanning using a spinning disk microscope. The time interval was at 2.64 sec. *Arrows* indicate the circular F-actin bundles in the root hairs. The images revealed that the circular F-actin bundles showed dynamic changes in the root hair. Bar = 5 µm.

**Figure 5:**
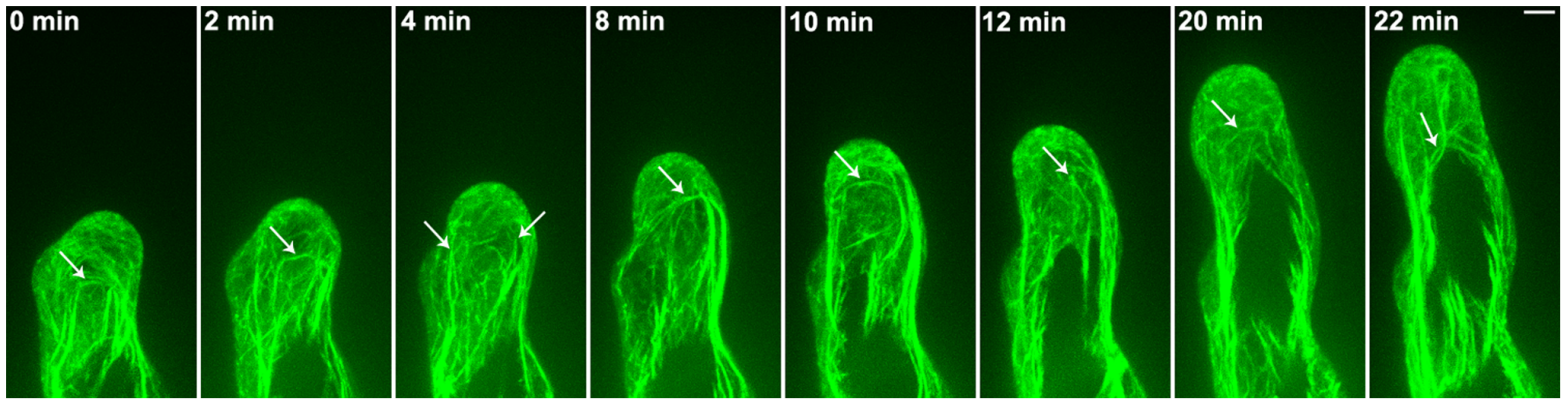
Confocal images of the circular F-actin bundles in growing root hair of *Arabidopsis.* The confocal images were obtained for time-lapse scanning using a spinning disk microscope. The time interval was at 2 min. These images revealed that the circular F-actin bundles existed in growing root hair. *Arrows* indicate the circular F-actin bundles in sub-apex of growing root hair. Bar = 5 µm.

We used a spinning disk confocal microscope for the time-lapse scanning of circular F-actin bundles in root hairs. This process revealed that circular F-actin bundles not only were constantly present during the entire process of root hair growth but also underwent dynamic changes (Figure 4, Movie S1). During the majority of the growth process, circular F-actin bundles were largely aligned along both sides of the periplasm of root hairs; on other occasions, F-actin bundles migrated from the periplasm to surround root hair centers. Furthermore, we examined the distribution of F-actin bundles in the same root hair during root hair growth and found that circular F-actin bundles were continuously present within this hair (Figure 5).

### Circular F-actin Bundles Provide a Track for Turnaround Mitochondrial Movement in Root Hairs

**Figure 6:**
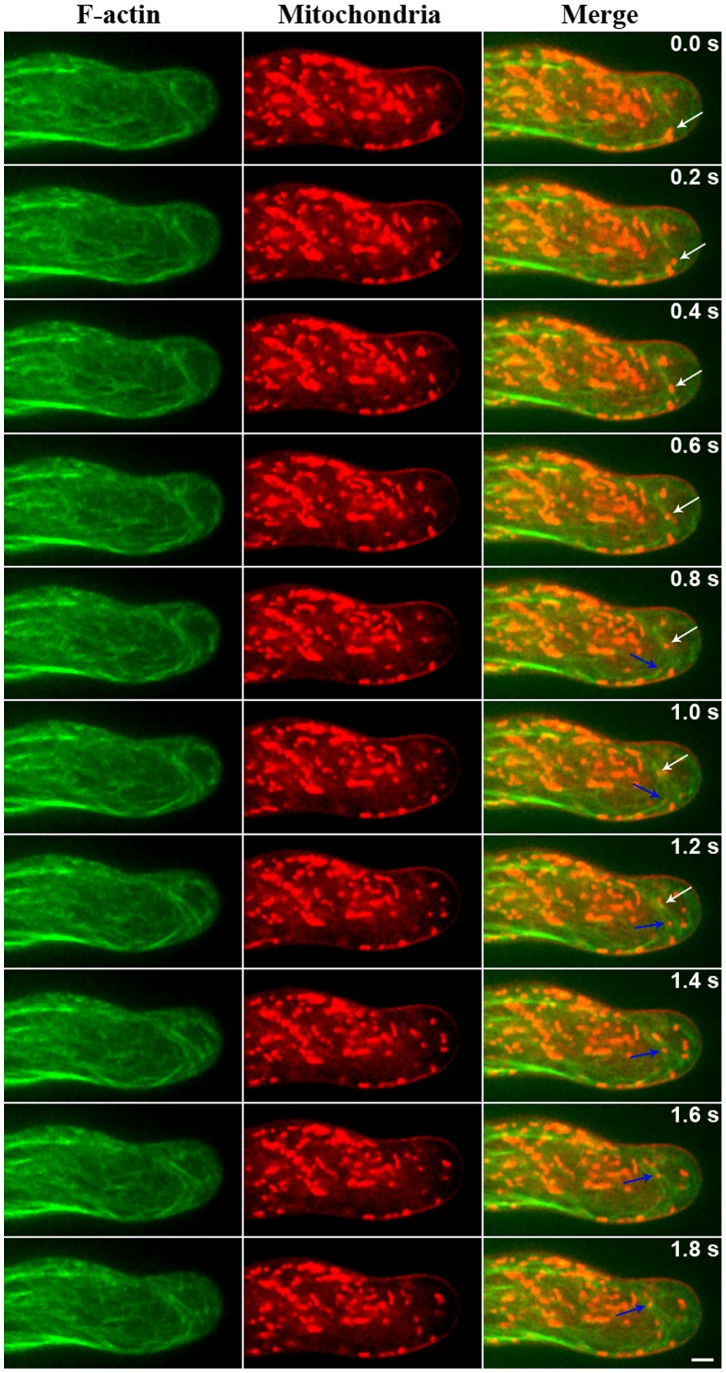
Mitochondria turn around along the circular F-actin bundles in sub-apical region of *Arabidopsis* living root hair. F-actin (green) in living root hairs were labeled by GFP-ABD2-GFP which was stable expression in *Arabidopsis*. Mitochondria (red) in root hair were stained by 2 nM MitoTracker Red CMXRos. The confocal images were obtained for time-lapse scanning using a spinning disk microscope. The time interval was at 0.2 sec. The white and blue arrows indicate two mitochondria respectively, of which turnaround was along the circular F-actin bundles at sub-apex of living root hairs. Bar = 5 µm.

To further elucidate the role of circular F-actin bundles, we used MitoTracker to label mitochondria in *Arabidopsis* root hairs and obtained time-lapse observations of microfilaments and mitochondria in root hair cells with a spinning disk confocal microscope. These observations revealed that the majority of mitochondria moved along longitudinal F-actin bundles in *Arabidopsis* root hair cells. In addition, we detected a few turnaround mitochondrial motions along the circular F-actin bundles in the sub-apical regions of root hairs (Figure 6, Movie S2). Three different movement types of mitochondria were observed in the sub-apical regions of root hair in Movie S2. During a time interval of 16 s in this movie, 19 mitochondria turned around on the F-actin loop, 11 mitochondria reversed direction on the straight F-actin cables, and 12 mitochondria reversed direction with no obvious associated F-actin bundles.

## Discussion

At present, fusion proteins that combine a fluorescent protein with an actin-binding protein or the actin-binding domain of this protein are frequently used as probes to label actin filaments and demonstrate the distribution and dynamic changes of these filaments in the living cells of plants. Wang et al. found that GFP-ABD2-GFP could label the microfilaments in various *Arabidopsis* tissues and cells more clearly than other fusion proteins [30]. We utilized an *Arabidopsis* line that stably expressed GFP-ABD2-GFP as the experimental material for this study, and our observations with a confocal laser scanning microscope clearly revealed the distribution of F-actin bundles in root hair cells.

Many studies have examined the distribution of actin filaments in root hair cells. In particular, fluorescent labeling has been used in conjunction with fluorescence microscopy or confocal microscopy to demonstrate that F-actin bundles are longitudinally aligned along the axis of elongation of root hair cells. However, there remain conflicting perspectives regarding the distribution of actin filaments in the apical and sub-apical regions of root hairs. Using S1 labeling (i.e., labeling with myosin subfragment-1) of actin and observations with a transmission electron microscope, Tominaga et al. discovered that the arrowheads in the F-actin bundles in the transvacuolar strand and those in the sub-cortical region had opposite polarities. The direction of the arrowheads was acropetal in the transvacuolar strand and basipetal in the sub-cortical region [29]. However, to date, the way in which this bidirectional arrangement of F-actin bundles in root hair cells forms has remained unknown. Using homozygous *Arabidopsis* seedlings that stably expressed GFP-ABD2-GFP, we observed F-actin bundles in root hair cells and found that circular F-actin bundles were universally present in root hairs. We believe that these circular F-actin bundles provide the mechanism by which F-actin bundles are longitudinally aligned in two opposite directions along root hairs. Moreover, we found that at most stages of root hair growth, these circular F-actin bundles in root hairs are primarily found on both sides of the periplasm, with a small quantity of bundles that migrate from the periplasm to surround central regions. On the other hand, F-actin in a bundle was discovered to have uniform polarity in root hairs [29]. Given the changes of polarity in circular F-actin bundles, we believe that in addition to polarity differences in actin bundles between the periplasm region and the central area of growing root hairs, the F-actin bundles on the two sides of the periplasm of a root hair should also exhibit opposite polarities due to these continuous circular F-actin bundles.

In addition, circular F-actin bundles were present during various stages of root hair growth, suggesting that these bundles could play an important role in the growth of the apical region of root hairs. Furthermore, in a previous study, we detected the presence of circular F-actin bundles in pollen tubes; we believe that these circular bundles provide the mechanism by which F-actin filaments form bidirectional bundles in pollen tubes [31]. Based on all of the aforementioned results, we believe that circular F-actin bundles are universally present in the plant tip-growth cells and account for the bidirectional distribution of actin bundles along the longitudinal axis of cells.

Studies have indicated that organelles within plant cells primarily move along F-actin bundles. Furthermore, it has been observed that organelle movement within root hairs is mainly bidirectional along the longitudinal axis of root hair cells, occurring through circulatory or reverse fountain streaming. Because the distribution of circular F-actin bundles is similar to the track of organelle movements in root hair cells, we believe that circular F-actin bundles constitute the track along which the turnaround and bi-directional movement of organelles in these cells occurs. To test our hypothesis, we used fluorescent labeling to observe mitochondria in *Arabidopsis* root hairs and found that mitochondria completed a rapid turnaround movement along the circular F-actin bundles in root hair cells. This result suggested that circular F-actin bundles are indeed the track along which the movements of mitochondria and other organelles are turn around in root hairs. In addition, the existence of different movement types of mitochondria in the sub-apical regions of root hairs suggests that multiple mechanisms of mitochondria transport might underlie the reverse-fountain movement in the sub-apical regions of root hairs.

It is well known that the movement of organelles along the actin cytoskeleton is myosin-driven. The actin cytoskeleton promotes organelle movement within plant cells by working in combination with myosin [2], [3]. Avisar et al. demonstrated that in tobacco leaf cells, myosin XI-K was involved in the rapid trafficking of mitochondria [21]. Peremyslov et al. proved that *Arabidopsis* myosin XI-K and myosin XI-2 played important roles in mitochondria transport [27]. These results indicate that mitochondria transport is driven by myosin in plant cells. Based on these results and our own finding, we suggest that the circular F-actin bundles may provide a track for myosin-driven turnaround and bidirectional movement of mitochondria and other organelles in plant tip-growth cells.

## Conclusions

Relevant experimental results demonstrated that circular F-actin bundles provide the track for myosin-driven turnaround and bidirectional movement of mitochondria.

## Materials and Methods

### Plant Materials and Growth Conditions

*Arabidopsis* seeds from plant expressing the 35S::GFP-ABD2-GFP construct (provided by Elison B. Blancaflor) were surface-sterilized for 8 min in 0.5% (v/v) NaClO and then washed five times with sterilized distilled water. Subsequently, the seeds were germinated on a thin layer of solid medium containing one-half Murashige and Skoog salt, 1% sucrose and 1.2% agar at a pH level of 5.8 (1/2 MS medium) in Petri dishes under a 16-h light/8-h dark cycle at 22°C. 4- or 5-day-old seedlings were transferred to a laser scanning confocal Petri dish that was modified into a small rounded chamber with coverslips on its bottom. The chamber was filled with the same liquid medium but without agar, and the roots were covered by the same solid medium which was cut to 8 mm×8 mm square by a scalpel. It was paid close attention that the Petri dish and the scalpel were surface-sterilized in 75% ethanol for 5 min and then washed three times with sterilized distilled water. Seedlings were grown in the Petri dish – under the same condition as before for 24–36 h. During this period, the seedlings stabilized root growth and proceeded in the formation of new root hairs [32].

### Staining of Mitochondria

MitoTracker Red CMXRos (Invitrogen, M7512) was prepared as a 1 mM (w/v) stock solution in anhydrous dimethylsulfoxide (DMSO) at –20°C. The stock solution was diluted to a final concentration of 2 nM with 1/2 MS liquid medium. Mitochondria in root hairs were stained by 10 µL 2 nM MitoTracker Red CMXRos for confocal observation.

### Confocal Microscopy

Figure 1A, Figure 2B–D and Figure 3 were obtained using a Zeiss LSM 510 META confocal microscope, fitted with a 63×oil-immersion objective (numerical aperture 1.4). Other images (Figure 1B–E and Figure 2A) and all time series images were obtained using an Olympus IX81 inverted microscope equipped with a Yokogawa spinning-disc confocal head (Yokogawa Electric) and an Andor iXon charge-coupled device camera (Andor Technology), fitted with a 40× or 60× oil-immersion objective (numerical aperture 1.30 and 1.42). Root hairs expressing GFP-ABD2-GFP constructs was visualized using an excitation wavelength of 488 nm with fluorescence emission being captured between 505 nm and 530 nm. Mitochondria labeled by MitoTracker Red CMXRos in root hairs were visualized using an excitation wavelength of 561 nm with fluorescence emission being captured between 585 nm and 615 nm. Optical sections were obtained at the 0.45- to 2.50-µm step, but the time series images except Figure 5 were only single optical slice. Images analysis was carried out using the standard software supplied with the microscope or the software of Image J.

## Supporting Information

Figure S1 **Microscopic images of the GFP-ABD2-GFP stable expression roots and wild type roots of *Arabidopsis*.** (A) Bright field image of the GFP-ABD2-GFP stable expression root. (B) Fluorescent image of the GFP-ABD2-GFP stable expression root. (C) Bright field image of the wild type root. (D) Fluorescent image for the wild type root. The images were obtained using an Olympus SZX16 microscope with a DP72 cooled-CCD. Bar = 100 µm.
(TIF)

Movie S1 **The Dynamic circular F-actin bundles in growing root hair of *Arabidopsis*.** The movie revealed that the circular F-actin bundles showed dynamic changes in growing root hair. Bar = 5 µm.
(AVI)

Movie S2 **Mitochondria turn around along the circular F-actin in living root hair of *Arabidopsis*.** The movie revealed that the turnaround of a few mitochondria was along the circular F-actin bundles at sub-apex of living root hair. Bar = 5 µm.
(RAR)
