# Supplementary figures and images for: The Circular F-Actin Bundles Provide a Track for Turnaround and Bidirectional Movement of Mitochondria in Arabidopsis Root Hair
Source: PLoS One. 2014 Mar 13;9(3):e91501. doi: 10.1371/journal.pone.0091501 (PMC3953408; doi:10.1371/journal.pone.0091501)

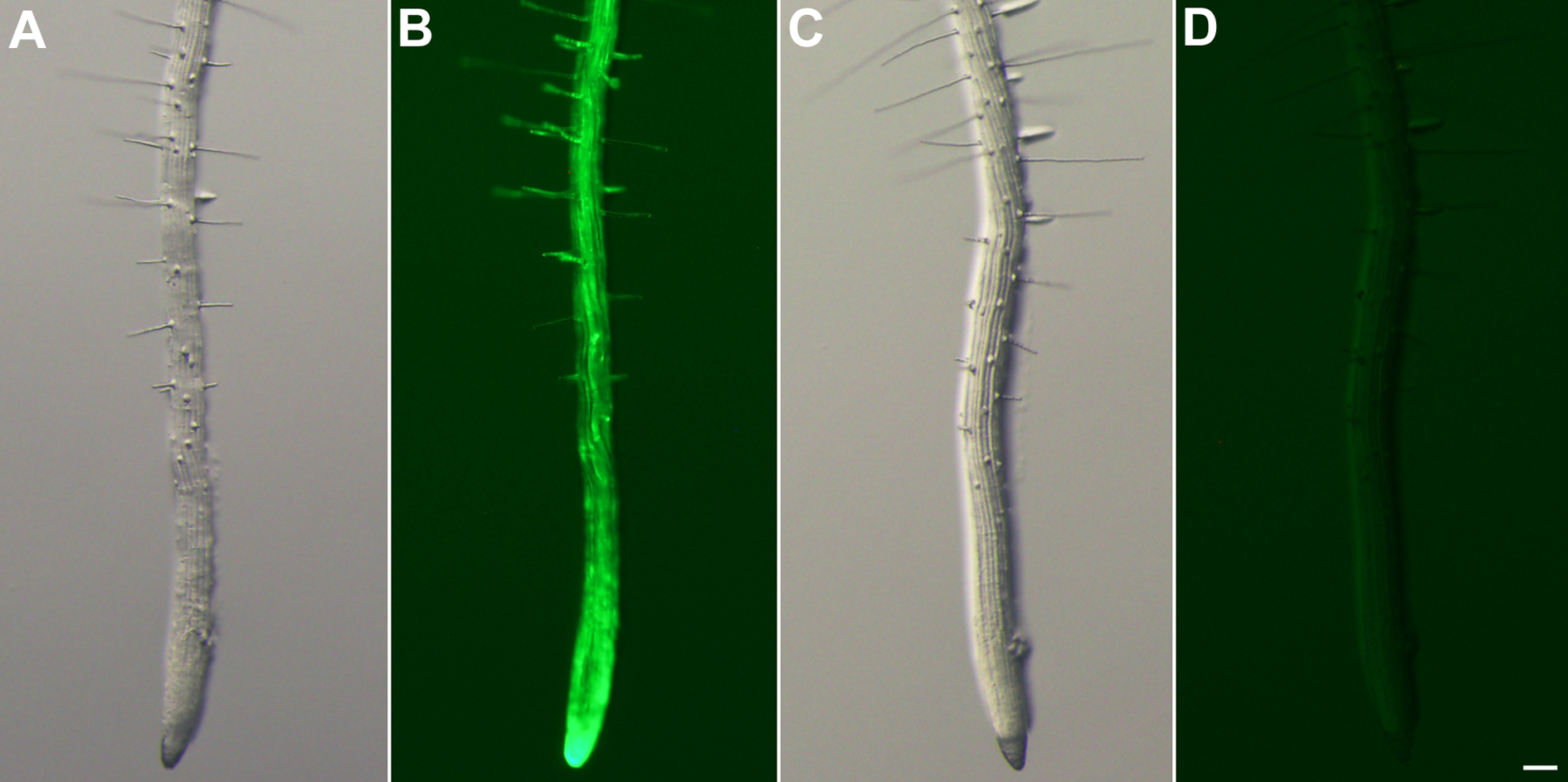

Supplement: Figure S1 — Microscopic images of the GFP-ABD2-GFP stable expression roots and wild type roots of Arabidopsis. (A) Bright field image of the GFP-ABD2-GFP stable expression root. (B) Fluorescent image of the GFP-ABD2-GFP stable expression root. (C) Bright field image of the wild type root. (D) Fluorescent image for the wild type root. The images were obtained using an Olympus SZX16 microscope with a DP72 cooled-CCD. Bar = 100 µm. (TIF) [file pone.0091501.s001.tif]
